# The active site structure and catalytic mechanism of arsenite oxidase

**DOI:** 10.1038/s41598-017-01840-y

**Published:** 2017-05-11

**Authors:** Thomas P. Warelow, M. Jake Pushie, Julien J. H. Cotelesage, Joanne M. Santini, Graham N. George

**Affiliations:** 1 Institute of Structural and Molecular Biology, Division of Biosciences, University College London, London, WC1E 6BT United Kingdom; 2 Department of Anatomy and Cell Biology, University of Saskatchewan, Saskatoon, SK S7N 5E5 Canada; 3 Molecular and Environmental Sciences Research Group, Department of Geological Sciences, University of Saskatchewan, SK, S7N 5E2 Canada; 4 Department of Chemistry, University of Saskatchewan, Saskatoon, SK S7N 5C9 Canada

## Abstract

Arsenite oxidase is thought to be an ancient enzyme, originating before the divergence of the Archaea and the Bacteria. We have investigated the nature of the molybdenum active site of the arsenite oxidase from the Alphaproteobacterium *Rhizobium* sp. str. NT-26 using a combination of X-ray absorption spectroscopy and computational chemistry. Our analysis indicates an oxidized Mo(VI) active site with a structure that is far from equilibrium. We propose that this is an entatic state imposed by the protein on the active site through relative orientation of the two molybdopterin cofactors, in a variant of the Rây-Dutt twist of classical coordination chemistry, which we call the pterin twist hypothesis. We discuss the implications of this hypothesis for other putatively ancient molybdopterin-based enzymes.

## Introduction

**Figure 1:**
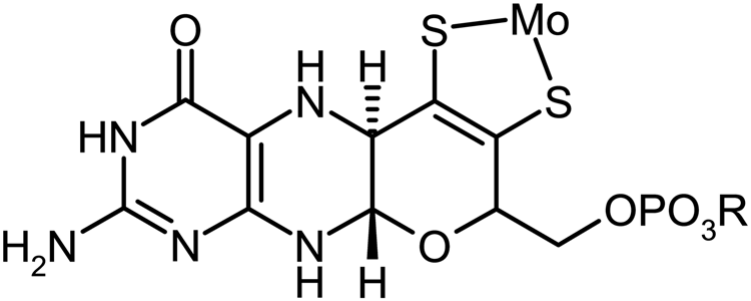
Schematic structure of the molybdopterin cofactor, the group *R* can be H, guanosine or cytosine, depending on the enzyme.

The group 6 transition metals molybdenum and tungsten are the only second and third row transition elements with known functions in biology^1^. Both metals are found in association with a novel pyranopterin-dithiolene cofactor called molybdopterin, with the metal coordinated by one or two of these through the ene-dithiolate moiety (Fig. 1). The enzymes constitute a distinct but widespread and evolutionary very ancient group, in that they originated prior to the split of the Archaea and Bacteria^1–4^. In most cases the tungsten enzymes are thought to have evolved before the molybdenum enzymes, and are believed to have been important in the last universal common ancestor^1, 5^, the progenitor of all known life. In almost all cases the enzymes catalyze reactions that involve two-electron oxidation-reduction chemistry coupled to the transfer of an oxygen atom to, or from, water^1–4^. The enzymes have been divided into three related families based on their active site structure, with by far the largest group being the dimethylsulfoxide (DMSO) reductase family^1–4^. We note in passing that the nitrogenase enzymes, are not formally members of the molybdenum and tungsten enzymes; while the most effective nitrogenases contain molybdenum, this enzyme has an active site that is unrelated to the molybdenum enzymes, and is placed in a unique category of its own^6^.

Arsenite oxidase (Aio) is a member of the DMSO reductase family of molybdenum enzymes that functions to oxidize arsenite to the less toxic arsenate:

$${\rm{As}}{({\rm{OH}})}_{3}+{{\rm{H}}}_{{\rm{2}}}{\rm{O}}\rightleftharpoons {[{\rm{As}}{({\rm{OH}})}_{2}{{\rm{O}}}_{2}]}^{-}+2{{\rm{e}}}^{-}+3{{\rm{H}}}^{+}$$

**Figure 2:**
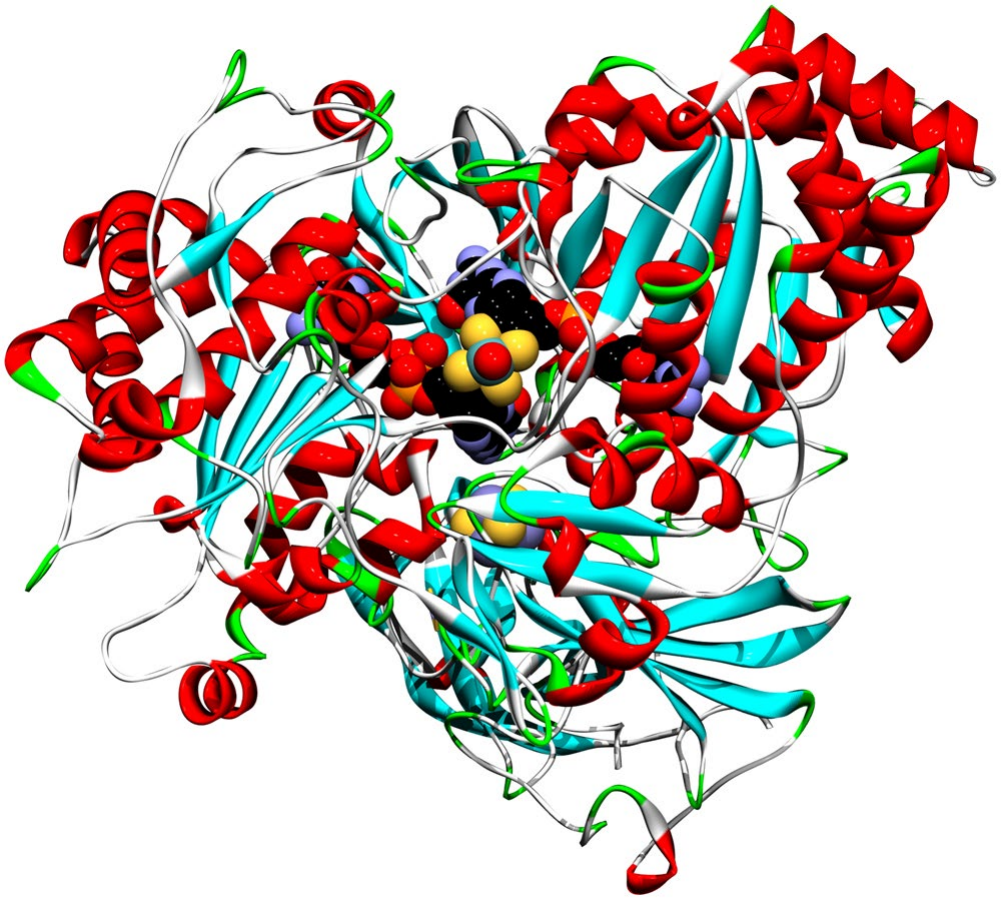
Ribbon representation of the crystal structure of *Rhizobium* sp. str. NT-26 arsenite oxidase^9^ showing the Mo site plus its two associated molybdopterin cofactors within the protein as CPK structures. The [3Fe-4S] cluster in the larger A subunit, and the Rieske [2Fe-2S] cluster in the smaller B subunit are also shown as CPK structures.

During the catalytic cycle the oxygen that is transferred to arsenite is thought to arise from an Mo = O group bound to molybdenum, and the molybdenum is reduced from the Mo(VI) to the Mo(IV) oxidation state. Arsenite oxidases from two representatives of different sub-phyla of the Bacteria (i.e., Alpha and Betaproteobacteria) have been structurally characterized by protein crystallography. The enzymes from *Alcaligenes faecalis* (betaproteobacterium)^7, 8^ and *Rhizobium* sp. str. NT-26 (alphaproteobacterium)^9^ show a number of common structural features, both being heterodimers with the molybdenum site and an [3Fe-4S] cluster in the larger A subunit, and a Rieske [2Fe-2S] cluster in the smaller B subunit (Fig. 2)^7, 9^. Both enzymes lack significant detectible Mo(V) EPR signals, and in agreement with this observation cyclic voltammetry of the *A. faecalis* enzyme shows an unusual highly cooperative two-electron Mo(IV)/Mo(VI) redox couple centred at 292 mV vs. SHE at pH 5.9^10^.

X-ray absorption spectroscopy (XAS) has been essential in our understanding of the active site structures of molybdenum enzymes as it provides structural details that crystallography alone often cannot provide^1, 3, 11^. Moreover, many molybdenum enzyme crystal structures are actually of photo-reduced forms^3, 12^ and because Mo K-edge XAS is much less prone to photo-reduction key information on the structure of the oxidized active sites often comes primarily from this technique^3^. We report herein a combined Mo K-edge XAS and density functional theory (DFT) study of the molybdenum active site of the NT-26 Aio and show that the enzyme possesses a novel *cis*-dioxo structure in the oxidized Mo(VI) form. We also show that this form of the oxidized enzyme must arise from a geometry that is far from the minimum energy for the Mo site and discuss the implications for the catalytic mechanism.

## Results and Discussion

### X-ray absorption spectroscopy

**Figure 3:**
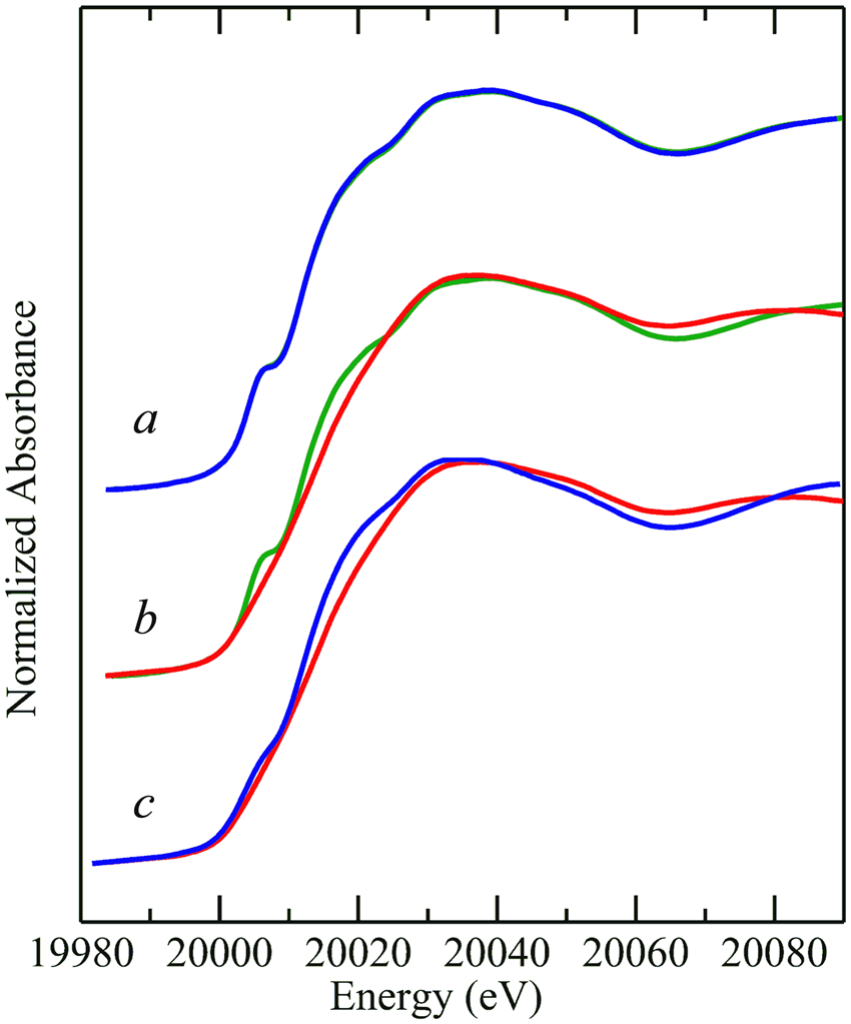
Mo K-edge X-ray absorption near-edge spectra of NT-26 arsenite oxidase. The as-isolated enzyme is shown in *a* () with the same form of the enzyme after addition of 5 mM arsenite (). The two traces overlay almost completely within the width of the line, showing that there are no significant changes in the Mo site arising from addition of substrate. The effects of addition of 5 mM (final) ferricyanide solution are shown in *b*, with the as-isolated protein () verses the ferricyanide-oxidized enzyme (). *c* shows the spectrum of ferricyanide-oxidized enzyme () compared with that of arsenite-reduced enzyme ().

Figure 3 shows the Mo K-edge near-edge spectra of enzyme in the as-isolated form, together with enzyme in the presence of an excess of the substrate arsenite. The near-edge spectra of as-isolated enzyme did not change appreciably in the presence of arsenite, in fact, the spectra of as-isolated and arsenite treated enzyme are sufficiently similar to overlay in the plot of Fig. 3a. The as-isolated enzyme resisted extensive efforts to reduce the Mo site, and even in the prolonged presence of an excess of dithionite (10 mM) in the presence of the mediator dyes benzyl viologen and methyl viologen the near-edge spectrum changed only very subtly; insufficiently to be consistent with conversion of the Mo present to a different redox state (not illustrated).

In previous work on DMSO reductase and related enzymes^13–15^ we have reported that redox cycling of the enzyme can change the active site structure from an inactive form to an active form. In the present case, because of the lack of any effect of substrate and other exogenous reductants on the Mo site we added the mild oxidizing agent ferricyanide (5 mM). Ferricyanide is frequently and conveniently used as a mild oxidizer for redox-active biological molecules because conversion of ferricyanide [Fe(CN)_6_]^3−^ to ferrocyanide [Fe(CN)_6_]^4−^ involves no changes in metal coordination. This resulted in a significantly modified near-edge spectrum (Fig. 3b) with a shift to higher energies, consistent with a relative oxidation. The near-edge spectrum did not change on removal of the excess ferricyanide by aerobic gel-filtration followed by re-concentration, indicating that the ferricyanide-oxidized enzyme was stable. Addition of an excess of arsenite (10 mM) to this ferricyanide-oxidized enzyme caused a modified near-edge spectrum with a shift to relative lower energy, consistent with the presence of a reduced Mo active site (Fig. 3c). This redox behavior differs from that of the *A. faecalis* enzyme, which can be readily reduced by arsenite in the as-isolated form^8^. A comparison of the near-edge spectra of the NT-26 and *A. faecalis* enzymes indicates that the spectrum of the NT-26 ferricyanide-oxidized enzyme is essentially identical to that of the as-isolated oxidized *A. faecalis* enzyme (not illustrated).

**Figure 4:**
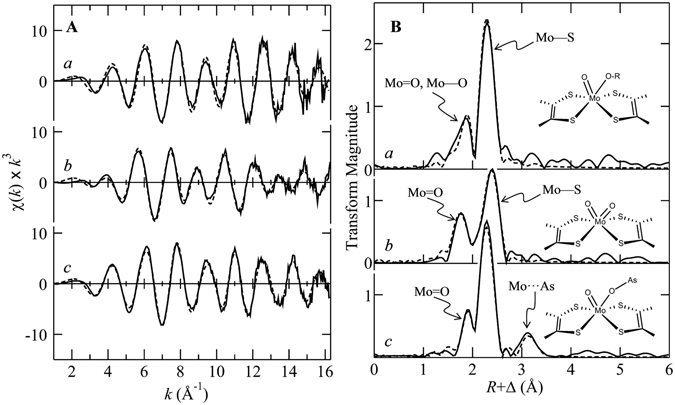
Mo K-edge EXAFS data (**A**) and corresponding Mo—S phase-corrected EXAFS Fourier transform (**B**). Solid lines () represent experimental data, and broken lines () the best fits, corresponding to the parameters given Table 1. *a* shows the data from as-isolated enzyme, *b* that from the ferricyanide oxidized Aio, and *c* that from enzyme reduced with excess arsenite. The insets in **B** show schematic structures of the molybdenum coordination sites.

The EXAFS of the different forms of NT-26 Aio investigated are shown in Fig. 4, together with the best fits and the corresponding Fourier transforms. The results of the EXAFS curve-fitting analyses are given in Table 1. In all cases, and as expected, the EXAFS data are dominated by intense backscattering from four Mo—S interactions arising from the two molybdopterin dithiolene ligands to the metal. The EXAFS of as-isolated enzyme shows an active site with four nearly equivalent Mo—S at 2.36 Å, one Mo = O at 1.73 Å, plus one Mo—O at 2.01 Å. Four Mo—S are expected from the *bis*-molybdopterin active site, a Mo = O was observed crystallographically, and the additional Mo—O is consistent with Mo—OH or other oxygen or nitrogen donor^16^.

**Table 1:** Summary of EXAFS curve fitting results^a^.

*Species*	Mo—O			Mo—S			Mo····As			*ΔE*_*0*_	*F*
	*N*	*R*	*σ* ^*2*^	*N*	*R*	*σ* ^*2*^	*N*	*R*	*σ* ^*2*^		
*As-isolated*	1	1.732 (3)	0.0017 (1)	4	2.358 (2)	0.0023 (1)				−16.6 (5)	0.281
	1	2.011 (5)	0.0023 (3)								
*Oxidized*	2	1.767 (2)	0.0031 (1)	4	2.457 (2)	0.0039 (1)				−15.8 (4)	0.227
*Arsenite-reduced*	1	1.746 (5)	0.0023 (1)	4	2.350 (2)	0.0029 (1)	1	3.25 (1)	0.0044 (4)	−16.9 (5)	0.232
	1	2.023 (6)	0.0033 (3)								

^a^Coordination numbers, *N*, interatomic distances *R* (Å), Debye-Waller factors *σ* ^*2*^ (Å^2^), and threshold energy shift Δ*E*_*0*_ (eV). The fit error parameter *F* is given by $F=\sqrt{{\sum }^{}{k}^{6}{({\chi }_{exptl}-{\chi }_{calc})}^{2}/{\sum }^{}{k}^{6}{\chi }_{exptl}}$, where ${\chi }_{exptl}$ and ${\chi }_{calc}$ are the experimental and calculated EXAFS, respectively, and the summations are over all data points included in the refinements. Values in parentheses are the estimated standard deviations obtained from the diagonal elements of the covariance matrix; these are precisions and are distinct from the accuracies which are expected to be larger (*ca* ±0.01–0.02 Å for *R*, and ±10–20% for both *N* and *σ* ^*2*^). We note that relative accuracies (e.g. comparing two different Mo—S bond-lengths) will be more similar to the precisions. The amplitude scale factor, otherwise known as the many-body amplitude reduction factor, or *S*_*0*_^*2*^, was defined by fitting data from a number of model compound species as 1.05. In all cases the *k*-range of the data fitted was from 1.0 to 16.2 Å^−1^.

The EXAFS bond-length resolution Δ*R* is defined as the minimum difference in distance to similar backscatterers that can be discerned. This is a function of the extent of the experimental data in *k*, and approximately given by Δ*R* ≈ π/2*k*, which relates to the *k* value at which a beat arising from the different EXAFS is visible. In the case of our data on NT-26 Aio, for which *k* extends to 16.2 Å^−1^, Δ*R* is a little less than 0.1 Å. In EXAFS analysis a simple Gaussian model ${e}^{-2{\sigma }^{2}{k}^{2}}$ for the pair distribution function of absorber-backscatterer pairs is employed, in which ${\sigma }^{2}$ is the mean-square deviation in absorber-backscatterer distance. Each ${\sigma }^{2}$ resulting from the EXAFS curve-fitting refinement has both vibrational and static components, with ${\sigma }^{2}={\sigma }_{vib}^{2}+{\sigma }_{stat}^{2}$, where ${\sigma }_{vib}^{2}$ arises from accessible vibrational states and ${\sigma }_{stat}^{2}$ arises from structural disorder in the bond-lengths differing by less than the EXAFS resolution Δ*R*.

1 $${\sigma }_{stat}^{2}\approx \frac{1}{n}{\sum ({R}_{i}-R)}^{2}$$

Where the difference between individual *R*_*i*_ and mean bond-length *R* $|{R}_{i}-R|\le \pi /2k$. As we have previously discussed^11^, ${\sigma }_{vib}^{2}$ can be computed and used to define a lower bound for *σ* ^*2*^. Assuming that no chemical heterogeneity is present in the sample, the value for ${\sigma }_{stat}^{2}$ computed from ${\sigma }^{2}-{\sigma }_{vib}^{2}$ can be used with eq. 1 to shed light on unresolved distribution bond-lengths^11^. In the case of the as-isolated NT-26 Aio, the Mo—S *σ* ^*2*^ value of 0.0023 Å^2^ is close to the value for ${\sigma }_{vib}^{2}$ which means that there is very little heterogeneity in the Mo—S bond-length. Similarly, the *σ* ^*2*^ values for the Mo = O and Mo—O ligands are close to their lower bounds^11^.

The ferricyanide-oxidized enzyme shows two very distinct peaks in the Fourier transform (Fig. 4). EXAFS curve-fitting shows that, like the as-isolated enzyme, the ferricyanide-oxidized Aio has four Mo—S backscatterers, but with a somewhat longer bond-length of 2.46 Å and a larger *σ* ^*2*^ value of 0.0039 Å^2^. This form of the enzyme also shows two Mo = O ligands at 1.77 Å, with a *σ* ^*2*^ value of 0.0031 Å^2^. The bond-length is consistent with a six-coordinate Mo(VI) *cis*-dioxo entity, but the *σ* ^*2*^ value is larger than the lower bound of ${\sigma }_{vib}^{2}$ for a Mo = O group of 0.0015 Å^2^ ^11^, and we estimate from this and eq. 1 that two different Mo = O bond-lengths are present at 1.73 and 1.80 Å. A similar, although slightly larger, disparity in Mo = O bond-lengths has been suggested on the basis of both vibrational spectroscopy and Mo K-edge XAS for the *A. faecalis* enzyme^8^. A number of structurally characterized *cis*-dioxo Mo(VI) small molecule species are known, with structures in the Cambridge structure database (CSD)^16^. These species are typically of octahedral type coordination around the Mo, with significant *trans*-effects such that there is elongation of the two Mo—S bonds *trans* to the two Mo = O ligands. Thus, examination of the CSD^16^ for six-coordinate *cis*-dioxo Mo(VI) species with four sulfur donors, shows mean Mo—S bond-lengths of 2.63 and 2.43 Å for Mo—S *trans* and *cis* to the Mo = O ligands, respectively. This bond-length difference of 0.2 Å would be very easily resolved in our EXAFS measurements and the Mo—S *σ* ^*2*^ value of 0.0039 Å^2^ indicates ${\sigma }_{stat}^{2}\approx 0.0016$ Å^2^ which is consistent with a Mo—S bond-length difference caused by Mo = O *trans*-influence of less than 0.06 Å. Thus, the active site of oxidized Aio, as revealed by XAS, is not consistent with octahedral type geometry.

The arsenite-reduced enzyme shows three discrete Fourier transform peaks, including a long-range peak at ~3.3 Å that was not observed in the other forms of the enzyme investigated. Curve-fitting analysis (Table 1) indicates four Mo—S backscatterers at 2.35 Å, a single Mo = O at 1.75 Å plus a longer Mo—O at 2.02 Å, and with an additional longer interaction that fits best to a Mo····As at 3.25 Å, suggesting the presence of bound substrate or product through a Mo—O—As linkage. We have previously observed similar Mo····As interactions in both DMSO reductase and sulfite oxidase^17, 18^. With DMSO reductase Mo····As EXAFS was observed in enzyme reduced with the product analog trimethylarsine with a somewhat longer Mo····As distance of 3.43 Å^17^, and with the arsenate complex of reduced human sulfite oxidase, which indicated a very similar Mo····As distance of 3.20 Å^18^. This form of the enzyme was generated using excess arsenite, and therefore the arsenic species is probably a product or a substrate complex. The fully reduced form of the enzyme did not form with stoichiometric arsenite, presumably because of intramolecular electron transfer to the iron sulfur centers, and this precluded experiments at the arsenic K-edge, which would have been able to unambiguously detect whether the complex is with substrate or product^17^.

### The structure of the oxidized active site

**Figure 5:**
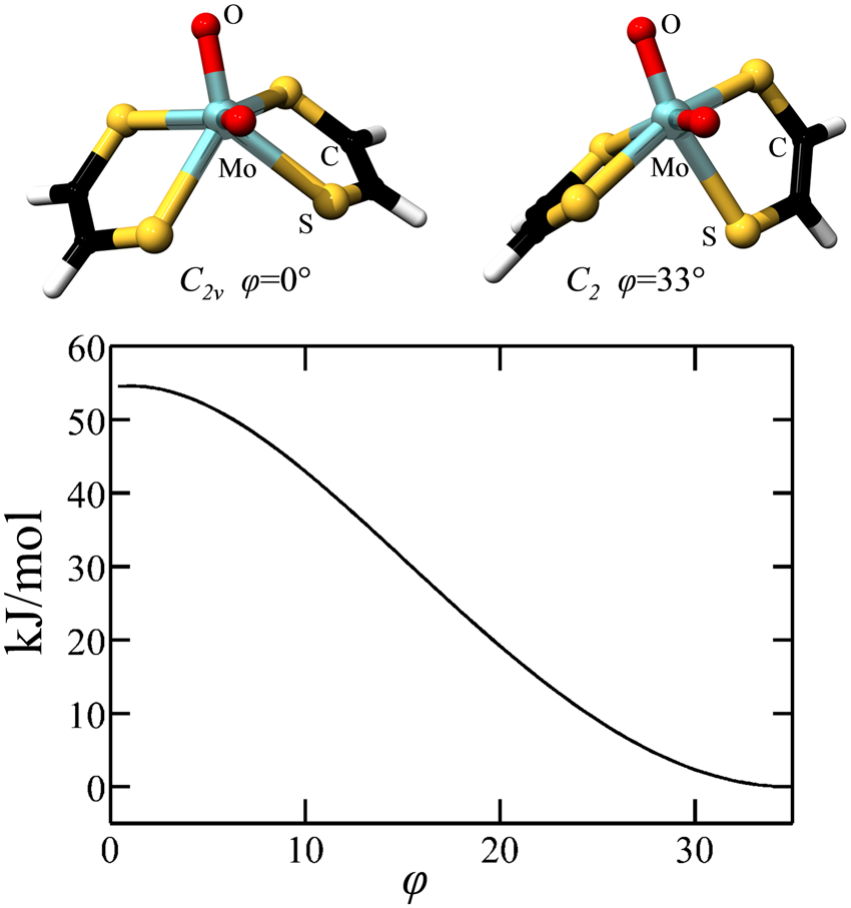
Idealized active site geometries as a function of the pterin twist angle *φ*, related to the Rây-Dutt twist of classical coordination chemistry. The structures show geometry optimized structures with enforced *C*_*2v*_ and *C*_*2*_ point group symmetry, and the DFT computed relative energies of the twist transformation is shown in the graph in the lower panel.

XAS provides accurate information related to the radial structure around a metal ion, but active site geometries are not directly available from this method. Fortunately, there are now a large number of crystal structures that provide this information and we can combine density functional theory (DFT) with XAS and crystallography to provide detailed insights^11^. There are very few examples among the Mo and W enzymes of pseudo-octahedral geometry in the active site. At the time of writing, the only such examples are acetylene hydratase^19^ and benzoyl CoA dehydrogenase^20^, both of which are W enzymes that catalyze reactions in a manner that is distinct from the mainstream Mo and W enzymes^19, 20^. For DMSO reductase family members, the typical geometry found is an approximate square based prismatic site, where the square base is formed by the four sulfurs of the two ditholenes of the molybdopterin cofactors. The molybdopterin cofactors represent large groups which typically fit in a distinct cleft within the protein. We have previously argued that because the cofactors are securely held within the protein that their orientation provides substantial constraints upon the orientation of the cofactor ligands to the metal, and that DFT energy minimized geometry optimized structures that are conducted without considering this can often be misleading^11, 21^. As we have discussed above, the XAS of the active site of oxidized Aio is not consistent with a pseudo-octahedral geometry equivalent Mo—S bonds. The DFT geometry optimized structures of an idealized octahedral and idealized square based prismatic geometries are shown in Fig. 5a, with respective point-group symmetries of *C*_*2*_ and *C*_*2v*_, respectively. The two structures are related by a twist of the dithiolene group, which can be defined as an angle *φ*, similar to the Rây-Dutt twist used in classical coordination chemistry to understand racemization of six-coordinate *tris*-chelate complexes^22, 23^. The angle *φ* varies from 0° for *C*_*2v*_ point-group symmetry through 35° for *C*_*2*_ point-group symmetry, and the two extreme geometries differ in energy by some 54 kJ/mol, as shown in Fig. 5b. According to the crystal structure, the enzyme active sites of both the *A. faecalis* and the NT-26 enzymes correspond to *φ* ≈ 15°, which places the active site at an energy which is at least 33 kJ/mol above the lowest-energy local geometry with octahedral-type *C*_2_ symmetry (Fig. 5b). We reiterate that the crystal structures^7, 9^ are in most cases thought to be of photo-reduced active sites^8^, and some subtle movement of the molybdopterin on oxidation may occur. We hypothesize that the oxidized active site exists in a non-equilibrium local geometry; an entatic state imposed by the protein on the active site through imposed relative orientation of the two molybdopterin cofactors. This would constitute an approach to transition state for oxidation of arsenite to arsenate, one of the classical expedients for catalysis^24^. We call this hypothesis the pterin twist hypothesis. Thus, XAS provides quantitative support for our hypothesis that, because of the pterin-twist effect, the *cis*-dioxo oxidized Mo(VI) active site geometrically resembles an idealized geometry for a reduced mono-oxo Mo(IV) site.

### DFT of calculations of active site and the catalytic process

**Figure 6:**
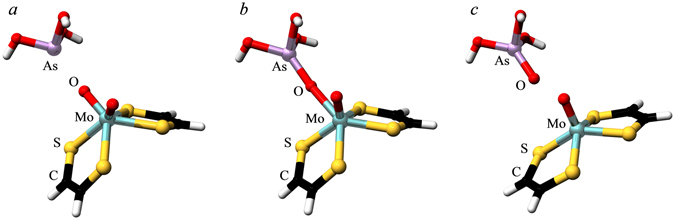
DFT transition state search. (**a**) Shows the starting model, based on the *C*_*2v*_ idealized active site geometry, with a molecule of *C*_*3v*_ arsenite in proximity to one of the Mo = O groups. The end product is shown in (**c**), which assumes a *C*_*2v*_ active site geometry similar to that observed crystallographically with arsenite (*C*_*3v*_) Both (**a** and **c**) were DFT geometry optimized structures with enforced local symmetry and constraining the Mo As distance. Constraints were lifted for the transition state search, the results of which are shown in (**b**).

**Figure 7:**
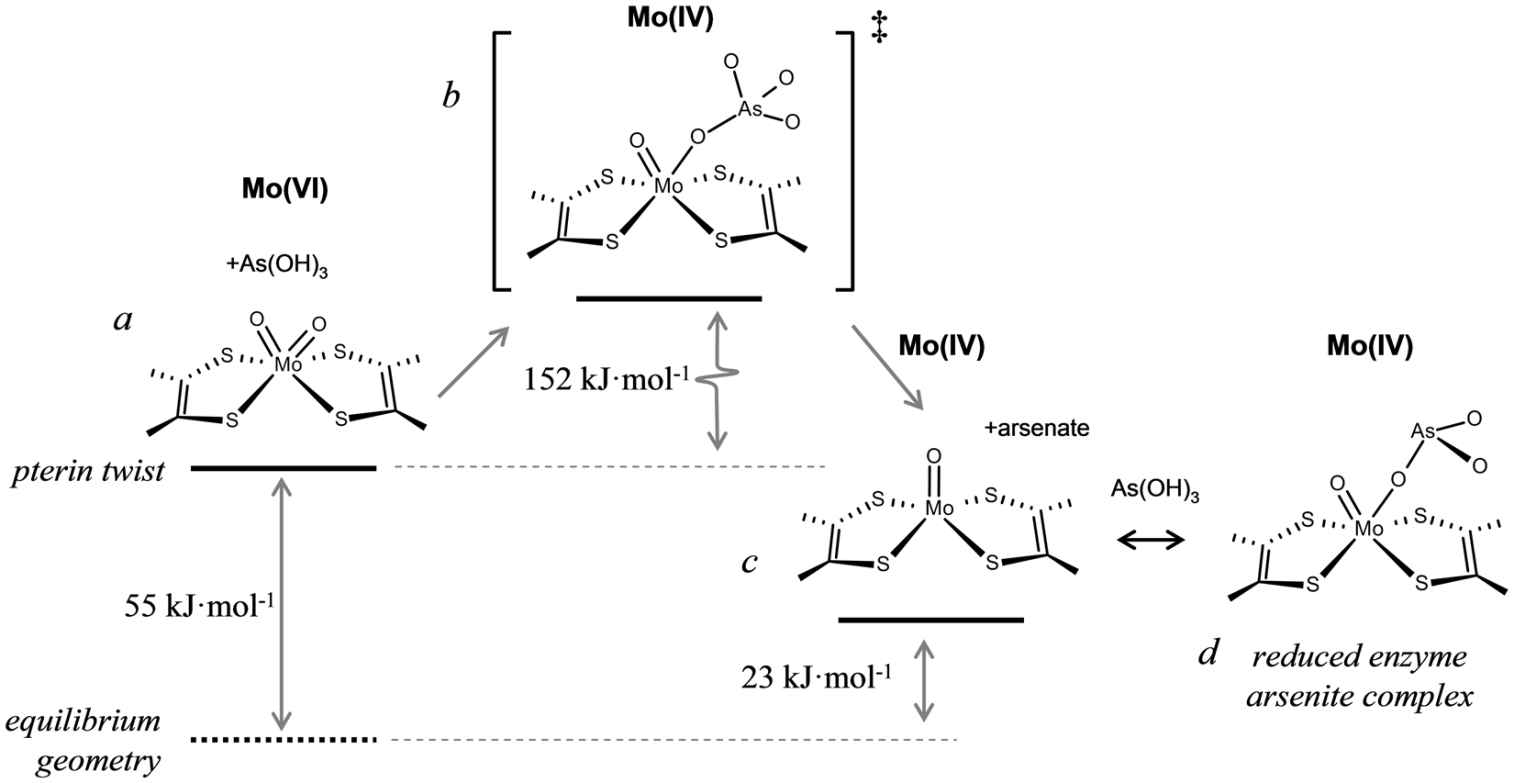
Schematic diagram of postulated reaction sequence for Aio. The initial *cis*-dioxo Mo(VI) site (**a**) is destabilized by some 55 kJ/mol from the pterin-twist induced by the protein, and reacts with arsenite in the active site pocket via a transition state (**b**) containing arsenic bound to molybdenum via a linking oxygen. This then breaks down to form the reduced Mo(IV) enzyme site plus the reaction product arsenate, (**c**). The Mo(IV) form observed with bound arsenic in the presence of excess substrate (arsenite) is also shown in (**d**), which we suggest to be a dead-end complex of reduced enzyme and arsenite. The oxidized active site is regenerated from (**c**) through intramolecular electron transfer to the other redox active centers, and reaction with solvent water (not shown) to form the *cis-*dioxo active site *a*, completing the catalytic cycle.

Figure 6 shows the results of a DFT transition state search using the *C*_*2v*_ oxidized Mo(VI) site structure of Fig. 5 as a starting point, with arsenite positioned near to one of the Mo = O groups (Fig. 6a). The end point of the reaction is shown in Fig. 6c, in which arsenate is located adjacent to a Mo(IV) site that resembles the reduced active site that has been observed crystallographically^7, 9^, with a local Mo symmetry approximating *C*_*2v*_, and the reduced active site observed for the *A. faecalis* enzyme^8^. The computed transition state is shown in Fig. 6b. It possesses an arsenic atom with four oxygens and close to tetrahedral geometry bound through oxygen to an active site that is intermediate between the oxidized and reduced sites. The Mo····As distance is 4.61 Å. The energetics from these calculations, together with schematic structures of the active sites are summarized in Fig. 7. The transition state is calculated to be 152 kJ·mol^−1^ above the oxidized starting point for the reaction, and the end point of the reaction 23 kJ·mol^−1^ below this energy. In agreement with the calculations shown in Fig. 6 the reaction start point is computed to be 55 kJ·mol^−1^ above the energy when the local Mo geometry symmetry restraints are relaxed to allow convergence on a local Mo site with effective *C*_*2*_ symmetry. Transition state searches starting from this fully relaxed *C*_*2*_-type Mo site structure proved reluctant to converge. The suggestion that protein can strongly influence active site properties is not without precedent. Perhaps the most well-known example is that of the blue copper proteins^25^ which have Cu(II) structures that are close to those expected for Cu(I), and with the Mo enzymes, Kirk and co-workers have suggested that the cysteine sulfur that ligates Mo in sulfite oxidase^26^ could be used by the enzyme to fine-tune the active site properties^26^.

### The structure of the reduced active site

The major difference between the EXAFS data of the NT-26 enzyme reported here, and that of the *A. faecalis* enzyme reported previously relates to the reduced enzyme. The *A. faecalis* data suggest a five-coordinate active site, with no discernable Mo····As EXAFS, whereas EXAFS of the NT-26 enzyme shows a more complex structure with a Mo····As at 3.25 Å, as discussed above. We hypothesize that this may be due to a dead-end substrate complex with reduced enzyme, as shown in Fig. 6. We have previously argued that constraints or restraints in DFT geometry optimization might be taken from crystallographic information^1, 3, 11^, and if this is done then we can compute a hypothetical reduced active site with bound arsenite, which has similar interatomic distances to those observed by EXAFS for NT-26 (Table 1, Fig. 4), indicating that the suggestion of a dead-end substrate complex with reduced enzyme is chemically reasonable.

### Relevance to other molybdopterin-based enzymes and significance of findings

Most of the other characterized members of the DMSO reductase family of Mo/W enzymes have a single amino acid side chain ligand to the Mo. This residue can be serine, cysteine, aspartate and selenocysteine, depending on the enzyme. To date, the only Mo enzyme in the DMSOR reductase family known to lack an amino acid side chain ligand to Mo is Aio. Nitschke and co-workers have used phylogenetic analysis to show that Aio probably evolved before the split of the Archaea and the Bacteria, which places it among the most ancient of the molybdopterin-based enzymes^27^. From a phylogenetic perspective the hyperthermophilic Archaea have been argued to be the most ancient organisms known^28^, although there is debate about this, and a non-hyperthermophilic common ancestor to life has also been postulated^29, 30^. Irrespective of whether or not a hyperthermophile was the ancestor of extant life, there is no debate that the hyperthermophilic Archea are very ancient organisms. The molybdopterin-based enzymes of the hyperthermophilic Archea appear to exclusively contain tungsten rather than molybdenum^1^. We have previously argued that tungsten may have preceded molybdenum because of different availabilities in the more reducing primordial oceans^1^, and thus the tungsten enzymes may be more ancient than the molybdenum enzymes. As a broad category, and irrespective of whether they contained Mo or W, the molybdopterin-based enzymes are thought to have filled a vital role in the biochemistry of early organisms because the predominantly O_2_-based mechanisms of modern biochemistry for oxygen atom transfer reactions were unavailable in the anoxic primordial environment^1^. Based on the limited data that is currently available, the molybdopterin-based hyperthermophilic archaeal tungsten enzymes resemble Aio in that they lack an amino acid to the metal^31–33^. Moreover, the EXAFS of the oxidized enzymes also indicates the presence of a *cis*-dioxo W(VI) active site with high sulfur coordination with no apparent *trans*-effects and close to homogenous W—S bond-lengths^34^, strikingly similar to that observed for Aio both in the present and previous work^8^, indicative of non-octahedral type coordination geometry. While, at the time of writing, our information is still incomplete regarding the metal site structures of these enzymes, we hypothesize that they share a common mechanism with Aio in tuning of the active site using a pterin twist. Irrespective of the possible role of the pterin twist mechanism in the W enzymes from hyperthermophilic Archaea, and of whether life’s origins lies with hyperthermophiles or otherwise, the fact that Aio is considered among the most ancient of the molybdopterin enzymes^27^ may mean that the pterin twist mechanism is fundamental to the earliest forms of these enzymes, and of significant importance to the earliest prokaryotic life, conducting otherwise difficult oxygen atom transferase chemistry.

## Methods

### Samples

Recombinant NT-26 Aio was prepared as previously described^9, 35^ as purified protein solutions with approximately 0.3 mM Mo. Activities of the purified protein were as previously described^9, 35^. XAS samples were prepared in 50 mM MOPS pH 7.5, frozen in 2 × 10 × 10 mm^3^ acrylic cuvettes and stored at liquid nitrogen temperatures until data acquisition.

### X-ray Absorption Spectroscopy data collection

X-ray absorption spectroscopy (XAS) measurements were conducted at the Stanford Synchrotron Radiation Lightsource (SSRL) with the SPEAR storage ring containing 500 mA at 3.0 GeV. Data acquisition used the program XAS Collect^36^. Molybdenum K-edge data were collected on the structural molecular biology XAS beamline 7–3, employing a Si(220) double-crystal monochromator. Beamline 7–3 is equipped with a rhodium-coated vertical collimating mirror upstream of the monochromator and harmonic rejection was accomplished by setting the mirror cutoff angle to 23 keV. Incident and transmitted X-ray intensities were monitored using nitrogen-filled ionization chambers using a sweeping voltage of 1.8 kV, and X-ray absorption was measured as the Mo K_α_ fluorescence excitation spectrum using an array of 30 germanium detectors^37^. Samples were maintained at a temperature of approximately 10 K during data collection using an Oxford instruments liquid helium flow cryostat. For each data set, between ten and twelve scans each of 40 min. duration were accumulated, and the energy was calibrated by reference to the absorption of a molybdenum foil measured simultaneously with each scan, assuming a lowest energy inflection point of 20003.9 eV. The energy threshold of the extended X-ray absorption fine structure (EXAFS) oscillations (*k* = 0 Å^−1^) was assumed to be 20025.0 eV.

### XAS data analysis

The EXAFS oscillations *χ*(*k*) were quantitatively analyzed by curve-fitting using the EXAFSPAK suite of computer programs^38^ as previously described^21, 39^, using *ab initio* theoretical phase and amplitude functions calculated using the program FEFF version 8.25^40, 41^. No smoothing, filtering or related operations were performed on the data.

### Density Functional Theory

Density Functional Theory (DFT) calculations employed Dmol^3^ BIOVIA Materials Studio 2016^42, 43^, using the Perdew-Burke-Ernzerhof functional^44, 45^ for both the potential during the self-consistent field procedure, and the energy. Dmol^3^ double numerical basis sets included polarization functions for all atoms, were spin-unrestricted, and all electron relativistic core treatments were used. Solvation effects were modeled by using the Conductor-like Screening Model (COSMO)^46^ with the dielectric constant of water (*ε* = 78.39) selected because of the solvent accessible nature of the active site. As we have previously reported^21^, the use of dielectric constants more typical of hydrophobic environments, (e.g. *ε* = 4.5) changes the DFT results only subtly, with bond-lengths to Mo typically changing by less than 0.005 Å and with relative energies of the different species calculated shifting very slightly, by less than 1 kJ/mol. Transition state searches were performed using the synchronous transit method within Dmol^3^ and Materials Studio, employing geometry-optimized reactants and products. A vibrational analysis subsequent to the transition state search showed that the derived transition state had a single imaginary vibrational frequency (corresponding to the reaction coordinate), with all other eigenvalues being real, confirming that a bona-fide transition state had been identified.
